# Born with a solitary kidney: at risk of hypertension

**DOI:** 10.1007/s00467-020-04535-1

**Published:** 2020-03-24

**Authors:** Claudio La Scola, Giuseppina Marra, Anita Ammenti, Andrea Pasini, Francesca Taroni, Cristina Bertulli, William Morello, Martina Ceccoli, Francesca Mencarelli, Stefano Guarino, Giuseppe Puccio, Giovanni Montini

**Affiliations:** 1 Nephrology and Dialysis Unit, Department of Pediatrics, Azienda Ospedaliero Universitaria Sant’Orsola-Malpighi, Bologna, Italy; 2 Pediatric Nephrology, Dialysis and Transplant Unit, Fondazione IRCCS Ca’ Granda – Ospedale Maggiore Policlinico di Milano, Milan, Italy; 3 Unità Polispecialistica Pediatrica, Ambulatorio Medi-Saluser, Parma, Italy; 4 Present Address: Department of Woman, Child and of General and Specialized Surgery, Università degli Studi della Campania L. Vanvitelli, Naples, Italy; 5 Palermo, Italy; 6 Giuliana and Bernardo Caprotti Chair of Pediatrics, Department of Clinical Sciences and Community Health, University of Milano, Milan, Italy

**Keywords:** Ambulatory blood pressure monitoring, Congenital solitary kidney, Hypertension, Children

## Abstract

**Background:**

Subjects with a congenital solitary kidney (CSK) are believed to be at risk of hypertension due to their low number of nephrons. However, as CSK is a congenital abnormality of the kidney or urinary tract (CAKUT), subtle dysplastic changes contributing to hypertension cannot be excluded.

**Methods:**

We retrospectively compared office blood pressure (OBP) and ambulatory blood pressure monitoring (ABPM) between two groups of children with CAKUT, aged 6–18 years: Group A with a CSK and Group B with two kidneys. All had normal renal parenchyma on scintigraphy and normal renal function. OBP and mean systolic and diastolic 24-h, daytime and nighttime ambulatory BP records were analyzed. The distribution of OBP and APBM as continuous values and the prevalence of hypertension (ambulatory/severe ambulatory or masked hypertension) in the two groups were compared.

**Results:**

There were 81 patients in Group A and 45 in Group B. Median OBP standard deviation scores were normal in both groups, without significant differences. Median ABPM standard deviation scores, although normal, were significantly higher in Group A and the prevalence of hypertension was higher (ambulatory/severe ambulatory or masked) (33.3 vs. 13.3%, *p* = 0.019), mainly because of the greater occurrence of masked hypertension.

**Conclusions:**

Our data show that a CSK per se can be associated with an increased risk of hypertension from the pediatric age. Therefore, ABPM, which has proved valuable in the screening of hypertension, is warranted in children with a CSK, even if laboratory and imaging assessment is otherwise normal.

**Electronic supplementary material:**

The online version of this article (10.1007/s00467-020-04535-1) contains supplementary material, which is available to authorized users.

## Introduction

Subjects with a congenital solitary kidney (CSK) are believed to be at risk of hypertension, due to their low number of nephrons. During the seventies, studies on animals with extensive renal ablation demonstrated glomerulosclerosis with progressive azotemia, proteinuria, and hypertension [1]. Since then, the association between a low number of nephrons and blood pressure dysregulation or hypertension has also been described in humans, in particular in subjects with a solitary kidney [2–11].

However, as CSK is a congenital abnormality of the kidney or urinary tract (CAKUT), subtle dysplastic changes undetected by imaging or standard laboratory assessment may also be present. Thus, hypertension may be secondary to either a low number of nephrons or to subtle dysplastic changes.

In order to ascertain whether CSK per se represents a risk factor for higher BP, we compared the BP values of children with CSK with those of children with CAKUT and two kidneys, as they both have, in principle, a similar probability of subtle dysplastic changes.

## Patients and methods

**Fig. 1:**
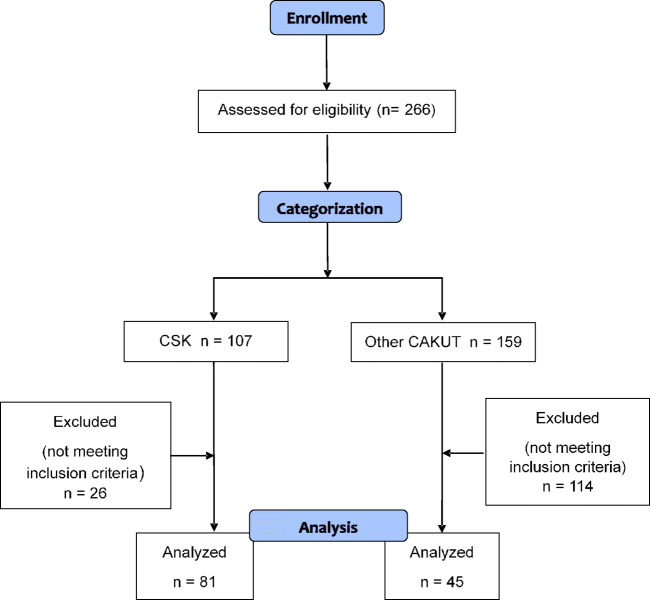
Flow-diagram of the studied population

In children aged 6 to 18 yrs. with CAKUT followed in two Pediatric Nephrology units in northern Italy, an ABPM was usually performed. Children with CAKUT who had undergone ABPM between January 2001 and December 2015 were considered for our retrospective analysis: there were 266 children altogether, 107 of whom had a CSK (Group A), and 159 had different types of CAKUT (Group B). From this population, eligible patients were selected according to the following inclusion criteria: OBP values available at the time of ABPM, a minimum of 80% of valid recordings at ABPM, normal kidney/kidneys morphology at ultrasound and Tc^99m^ dimercaptosuccinic acid or mercaptoacetyltriglycine scintigraphy (absence of renal scars, relative uptake in children with two kidneys between 45 and 55%), normal renal function as assessed by standard laboratory testing at the time of ABPM (estimated glomerular filtration rate with the original Schwartz formula > 90 ml/min/1.73m^2^, urinary protein/urinary creatinine < 0.2 mg/mg in random urine, first morning urine specific gravity ≥ 1020), and absence of heart or systemic diseases. Twenty-six patients (24%) were excluded from Group A, mainly because of insufficient valid readings at ABPM. From Group B, 114 patients (72%) were excluded due to abnormal scintigraphy or, less frequently, abnormal renal function; some of them also had insufficient readings at ABPM. Ultimately, 126 patients were eligible, 81 in Group A and 45 in Group B (Fig. 1). Informed consent was obtained from the children’s parents and the local ethics committees approved the study.

Measurements of OBP were performed by means of the auscultatory method, using an aneroid sphygmomanometer on the non-dominant arm; the 5th Korotkoff sound was recorded for diastolic blood pressure (DBP). Readings were taken three times after at least five minutes rest and the mean of the three measurements was recorded.

Values were transformed into Standard Deviation Scores (SDSs) and percentile category for sex and height, according to published normal values [12].

Ambulatory blood pressure monitoring was performed using an oscillometric device (Spacelabs Healthcare, model 90217), approved by the Association for the advancement of medical instrumentation and the British Hypertension Society [13, 14], and an appropriate cuff size on the non-dominant arm. Recordings were obtained over a period of 24 h. Blood pressure was measured 3 times an hour from 8 am to 8 pm, once an hour from midnight to 6 am and twice an hour from 6 to 8 am, and from 8 pm to midnight. Data collected between 8 am and 8 pm were analyzed as daytime blood pressure (BP), while data collected between midnight and 6 am were analyzed as nighttime BP, as suggested by Wühl et al. [15]. Mean BP levels, dipping, and load were analyzed as follows:

Twenty-four-hour, daytime and nighttime systolic BP (SBP) and DBP, mean arterial pressure (MAP) data, expressed as mean levels, were converted into SDSs and percentile category for height and sex, using the modified LMS method proposed by Cole and Green [16], which describes the distribution of a measurement Y by its median (M), the coefficient of variation (S), and a measure of skewness (L), required to transform the data to normality. For the computation, we used published reference LMS tables for healthy children, and the related equations [15].

Nocturnal dipping was expressed as a percentage of day/night difference ([mean awake BP-mean sleep BP]/mean awake BP × 100) for both SBP and DBP. Reduced dipping was defined as a drop ≤ 10% in nocturnal vs. daytime BP values [13].

BP load, which was only available in 79/126 patients, was expressed as the percentage of systolic or diastolic readings above the 95th percentile during the entire 24-h period, and was defined abnormal if ≥ 25% [13].

### Blood pressure evaluation

The distribution of individual values of OBP and APBM was analyzed in the two groups.

Moreover, by comparing mean office SBP and DBP with mean 24-h, daytime and nighttime ABPM data expressed as percentiles (< 90th, ≥ 90th < 95th, and ≥ 95th percentile), we classified BP into the following four categories:

1. Normal BP (OBP < 90th, ABPM < 95th percentile)
2. White coat hypertension/Pre-hypertension (OBP ≥ 90th, ABPM < 95th percentile)
3. Masked hypertension (OBP < 95th, ABPM > 95th percentile)
4. Ambulatory hypertension/Severe ambulatory hypertension (OBP > 95th, ABPM > 95th percentile).

Our categorization differs from that of the American Heart Association (AHA), which includes load, as data on load were only available in a subset of our patients. The main difference is that we combined white coat hypertension and pre-hypertension, which in the AHA scheme are separated according to load values [13]. Staging according to the AHA rules was performed as an additional analysis in the subset of patients for whom load data were available. The results are shown in Online Resource 1.

Hypertension as a binary variable was defined as the presence of ambulatory hypertension/severe ambulatory hypertension or masked hypertension.

### Outcomes

We considered the following outcomes in the two groups: differences in the continuous distribution of OBP and APBM values; differences in the prevalence of the above mentioned four BP classes, differences in the prevalence of hypertension, differences in the prevalence of reduced nocturnal dipping, and abnormal load.

### Patient data

The following data, collected at the time of ABPM, were retrieved: demographic and medical history (sex, gestational age, birth weight); clinical data (height, weight, body mass index); laboratory values (glomerular filtration rate estimated by the original Schwartz formula); imaging (ultrasound renal length, type of CAKUT).

### Risk factor analysis

We analyzed whether the following risk factors were associated with the primary outcome in the CSK group: age, sex, gestational age, prematurity (< 37 weeks gestational age), birth weight, low birth weight (< 2500 g), obesity (body mass index ≥ 95th percentile for age and sex), vesico-ureteric reflux, renal hypertrophy (ultrasound length of CSK above 95th percentile for normal kidneys) [17], eGFR values, hyperfiltration (which we defined as eGFR higher than the mean for age + 1 SD, according to normal published values for CSK) [18].

### Statistical analysis

Statistical analysis was performed using the open source software R [19]. The Chi-Square test of independence and Fisher’s exact test were used to analyze the relationship between categorical variables.

Non-parametric tests (Wilcoxon, Kruskal-Wallis) were used to analyze the difference in the distribution of a continuous variable in two or more different groups. ABPM values were transformed into SDS scores for height and sex according to published references for the general population, using the procedure described in detail in the section “Blood pressure measurements and evaluation”.

A correction for multiple comparisons was applied, where appropriate, according to Holm’s method.

## Results

Of the 126 children included in the study, 81 with CSK (agenesis 32, multicystic dysplastic kidney 30, aplasia 10, undefined 9) were assigned to Group A; Group B consisted of 45 children with other types of CAKUT (vesico-ureteric reflux 22, uretero-pelvic junction obstruction 7, uretero-vesical junction obstruction 2, posterior urethral valves 6, urinary tract dilatation 6, other 2). The demographic and clinical data of the two groups are shown in Table 1. Median age at ABPM (11.8 vs. 14.2 yrs., *p* = 0.002) and rate of vesico-ureteric reflux (18% vs. 49%, *p* = <0.001) were significantly different between the two groups, while no significant differences were observed in the other parameters.

**Table 1:** Demographic and clinical data of 81 children with CSK (Group A) and 45 children with two kidneys and other types of CAKUT (Group B)

Demographic and clinical data	Group A (*n* = 81)	Group B (*n* = 45)	*p* value
Age, median (IQR), years	11.8 (4.7)	14.2 (3.6)	0.002
Male sex	61/81 (75.3%)	34/45 (75.6%)	0.975
Height Z score, median (IQR)	− 0.1 (1.7)	− 0.3 (1.1)	0.969
Body mass index Z score, median (IQR)	0.6 (1.0)	0.1 (1.6)	0.067
Obese	4 (4.9%)	6 (13.3%)	0.343
Gestational age, median (IQR), weeks	39 (3)	39 (2)	0.207
Preterm birth	10/58 (17.2%)	2/22 (9.1%)	0.362
Birth weight, median (IQR), grams	3082 (712)	3200 (500)	0.303
Low birth weight	9/56 (16.1%)	0/21(0.0%)	0.051
Vesico-ureteric reflux	9/50 (18%)	22/45 (50%)	0.000
eGFR median (IQR), ml/min/1.73m^2^	122.4 (33)	124.4 (33)	0.350

CSK, congenital solitary kidney; CAKUT, congenital abnormalities of kidney and urinary tract; IQR, interquartile range; eGFR, estimated glomerular filtration rate

### Outcomes

#### Office blood pressure

Median office SBP and DBP SDS were normal in both groups, and no significant differences between them were observed (Table 2); the same was true when patients were classified according to their OBP percentiles (Table 3).

**Table 2:** Office BP and ABPM parameters expressed as medians in children with CSK (Group A) and children with two kidneys and other types of CAKUT (Group B)

	Group A (*n* = 81)	Group B (*n* = 45)	*p* value	*adjusted p (Holm’s method)*
	*Median [Range (IQR)]*	*Median [Range (IQR)]*		
Office BP Parameters				
SBP_SDS_	0.40 [− 1.9 • 4.2 (1.2)]	0.04 [− 1.5 • 2.3 (1.2)]	0.1186	0.4744
DBP_SDS_	0.19 [− 1.5 • 2.5 (0.8)]	0.34 [− 1.4 • 1.7 (0.8)]	0.6358	0.9332
ABPM Parameters				
24-h SBP_SDS_	0.6 [− 1.3 • 3.4 (1.3)]	− 0.1 [− 1.9 • 3.3 (1.1)]	0.0003	0.0052
Daytime SBP_SDS_	0.2 [− 1.8 • 2.9 (1.1)]	− 0.15 [− 1.9 • 2.6 (1.2)]	0.0089	0.0623
Nighttime SBP_SDS_	1.1 [− 1.1 • 3.9 (1.3)]	0.5 [− 1.4 • 5.6 (0.8)]	0.0017	0.0150
24-h DBP_SDS_	0.2 [− 1.4 • 3.5 (1)]	− 0.3 [− 2.2 • 3.2 (1)]	0.0008	0.0095
Daytime DBP_SDS_	− 0.2 [− 1.8 • 2.4 (0.9)]	− 0.7 [− 2.0 • 1.3 (1.1)]	0.0095	0.0623
Nighttime DBP_SDS_	0.6 [− 1.2 • 4.1 (0.9)]	0.1 [− 1.2 • 7.1 (0.9)]	0.0010	0.0099
24-h MAP_SDS_	0.2 [− 1.6 • 4.7 (0.7)]	− 0.04 [− 2.0 • 2.6 (0.3)]	0.0009	0.0099
Daytime MAP_SDS_	0.1 [− 1.6 • 3.7 (1.1)]	− 0.4 [− 1.9 • 1.8 (1.1)]	0.0049	0.0396
Nighttime MAP_SDS_	0.7 [− 1.2 • 3.8 (0.9)]	0.3 [− 1.6 • 3.6 (0.8)]	0.0006	0.0091
Systolic Dipping%	8.3 [− 2.9 • 16.8 (5.1)]	9.4 [− 11.7 • 19.5 (7.9)]	0.4666	0.9332
Diastolic Dipping%	16.2 [1.5 • 30.6 (8.6)]	18.2 [− 22.5 • 33.3 (7.6)]	0.1839	0.5517
Systolic Load%	21.7 [0 • 87 (33)]*	8 [0 • 88 (11.7)]**	0.0251	0.1253
Diastolic Load%	17 [0 • 88 (27)]*	1 [0 • 74 (7)]**	0.0007	0.0091

*Calculated in 61/81 patients

**Calculated in 18/45 patients

BP, blood pressure; ABPM, ambulatory blood pressure monitoring; CSK, congenital solitary kidney; CAKUT, congenital abnormalities of kidney and urinary tract; IQR, interquartile range; SBP, systolic blood pressure; DBP, diastolic blood pressure; MAP, mean arterial pressure; SDS, standard deviation score

**Table 3:** Office BP and ABPM parameters in children with CSK (Group A) and children with two kidneys and other types of CAKUT (Group B) classified according to their percentile class

Office BP	Group A (n = 81)			Group B (n = 45)			*p* value	adjusted *p* (Holm’s method)
	< 90th%ile	≥ 90 < 95th%ile	≥ 95th%ile	< 90th%ile	≥ 90 < 95th%ile	≥ 95th%ile		
SBP	82.7%	3.7%	13.6%	91.1%	4.5%	4.4%	0.286	1.000
DBP	93.8%	3.7%	2.5%	95.6%	0%	4.4%	0.490	1.000
ABPM	Group A (*n* = 81)			Group B (*n* = 45)			*p* value	adjusted *p* (Holm’s method)
	< 95th%ile	≥ 95th%ile		< 95th%ile	≥ 95th%ile			
24-h SBP	81.5%	18.5%		95.6%	4.4%		0.027	0.297
Daytime SBP	86.4%	13.6%		95.6%	4.4%		0.106	0.954
Nighttime SBP	72.8%	27.2%		88.9%	11.1%		0.035	0.350
24-h DBP	96.3%	3.7%		97.8%	2.2%		0.649	1.000
Daytime DBP	98.8%	1.2%		100.0%	0.0%		0.454	1.000
Nighttime DBP	87.8%	12.3%		95.6%	4.4%		0.148	1.000
24-h MAP	92.6%	7.4%		97.8%	2.2%		0.223	1.000
Daytime MAP	93.8%	6.2%		97.8%	2.2%		0.318	1.000
Nighttime MAP	82.7%	17.3%		91.1%	8.9%		0.197	1.000

BP, blood pressure; ABPM, ambulatory blood pressure monitoring; CSK, congenital solitary kidney; CAKUT, congenital abnormalities of kidney and urinary tract; SBP, systolic blood pressure; DBP, diastolic blood pressure; MAP, mean arterial pressure

#### Ambulatory blood pressure monitoring

**Fig. 2:**
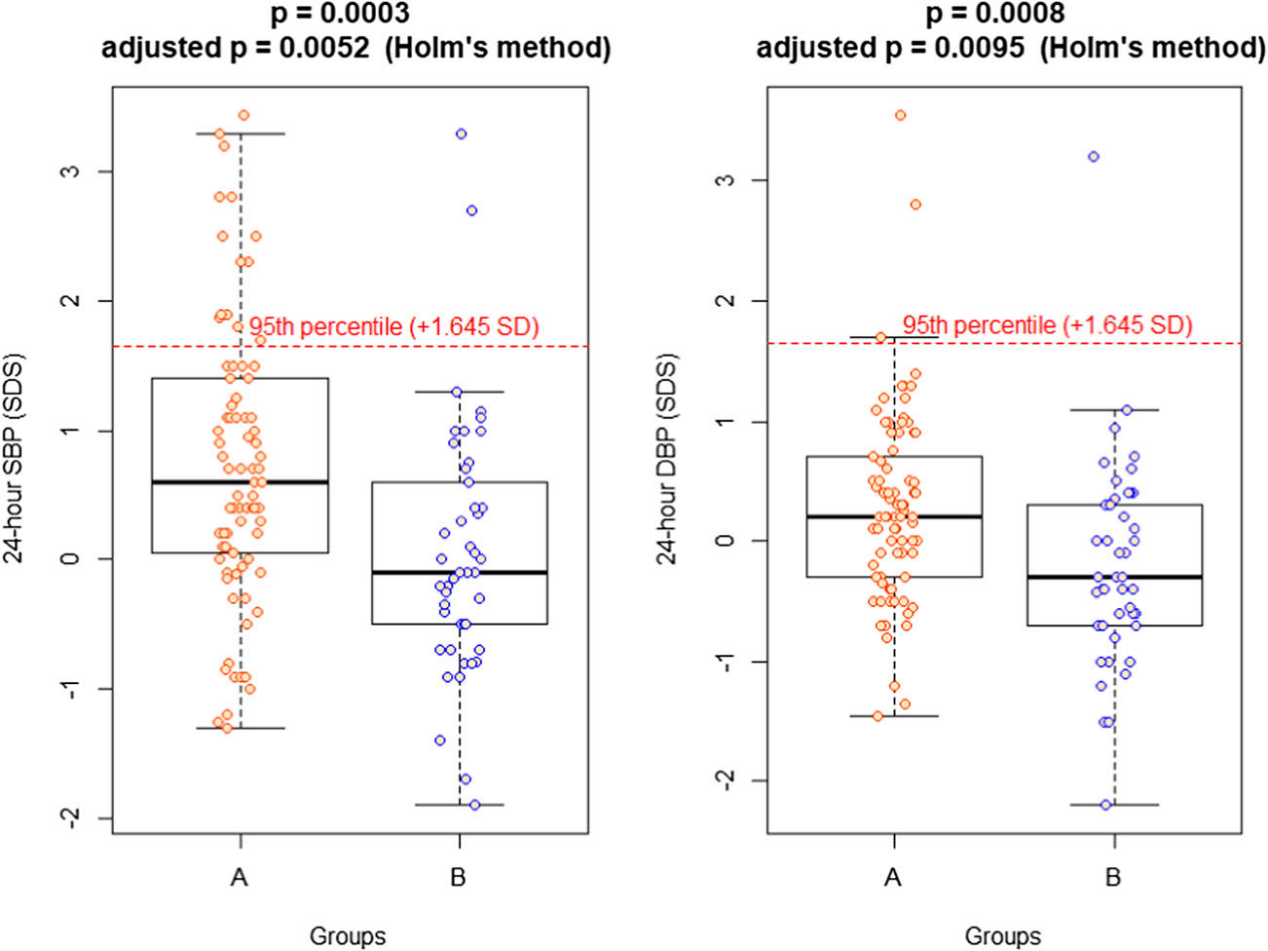
Boxplots of the distribution of 24-h SBP (SDS) and 24-h DBP (SDS) in the two groups

**Fig. 3:**
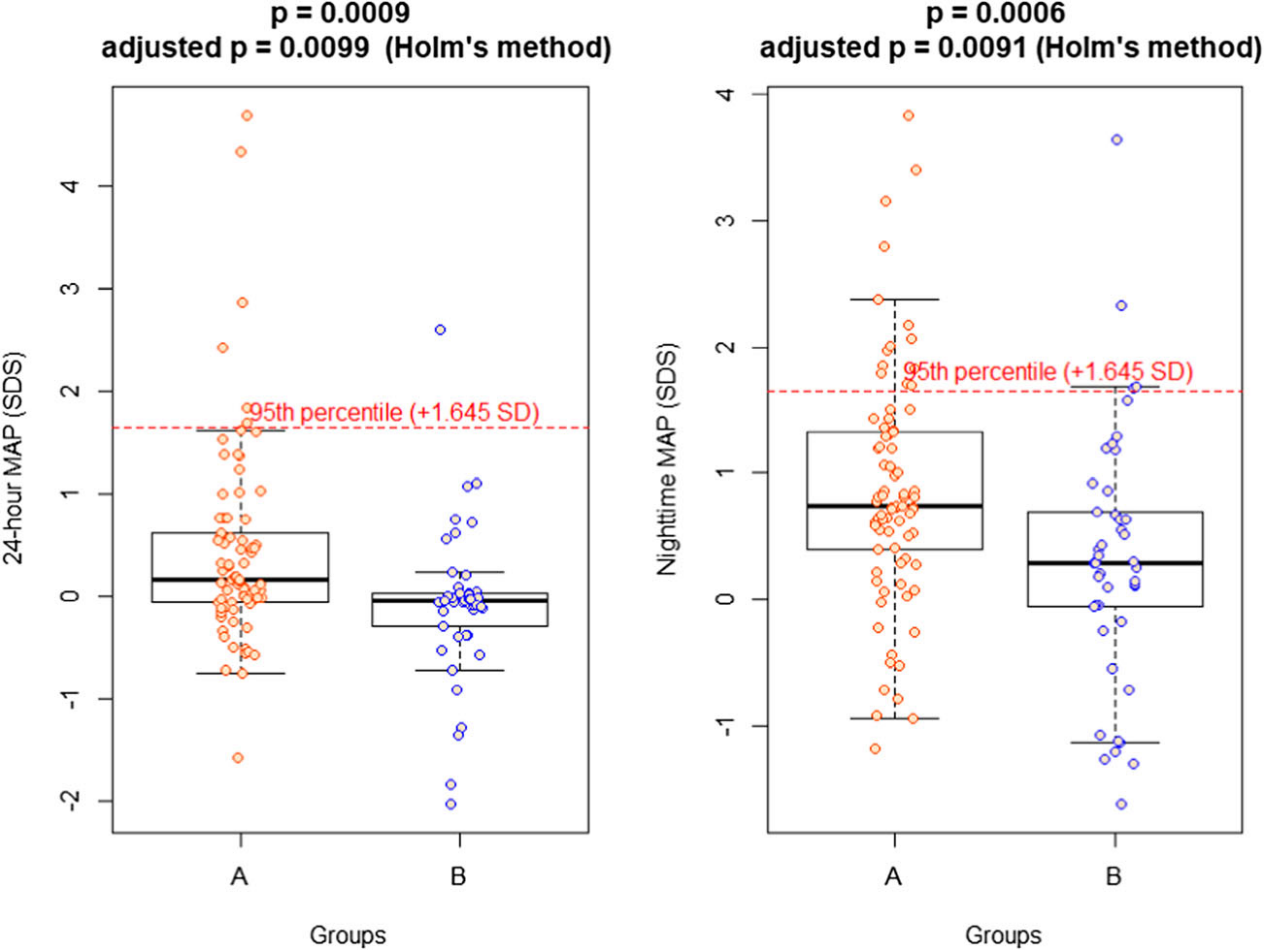
Boxplots of the distribution of 24-h MAP (SDS) and Nighttime MAP (SDS) in the two groups

Median ABPM SDSs were normal in both cohorts, but significantly higher in Group A for most parameters, even after Holm’s correction (Table 2). Figures 2 and 3 show the different distributions for the most significant parameters (24 h SBP and DBP, 24 hand nighttime MAP): it can be observed that the whole distribution is shifted to higher values in Group A.

Median systolic dipping was abnormal in both cohorts, without significant differences between the two groups. Systolic and diastolic load were calculated in 61/81 subjects in Group A and 18/45 in Group B. Median systolic and diastolic load was normal in both groups, but significantly higher in Group A, and the difference was extremely significant for diastolic values, even after Holm’s correction (Table 2).

When patients were classified according to their ABPM percentiles, the proportion of values ≥ 95th percentile was not significantly higher in Group A, except for weakly significant differences for 24-h SBP (*p* = 0.027) and nighttime SBP (*p* = 0.035). However, these differences were not significant after Holm’s correction (Table 3).

#### Blood pressure classes and hypertension

Taken as a whole, the differences in the distribution of the four diagnostic classes in the two groups were slightly significant (*p* = 0.047), with a lower prevalence of normal BP (58.0% vs. 82.2%) and a higher prevalence of the other three classes in Group A; in particular, masked hypertension was much higher in Group A (25.9% vs. 8.9%) (Table 4). Hypertension, defined as the occurrence of ambulatory/severe ambulatory or masked hypertension, was significantly higher in Group A (33.3% vs. 13.3%, *p* = 0.019). The subset of 79 patients for whom load values were available was divided into the 6 classes recommended by the AHA hypertension guidelines: the trend in the distribution of BP was very similar, especially for the prevalence of ambulatory and masked hypertension (Online Resource 1).

**Table 4:** Distribution of BP classes and prevalence of hypertension (ambulatory/severe ambulatory or masked) in children with CSK (Group A) and children with two kidneys and other types of CAKUT (Group B)

Classification	Group A	Group B	*p* value
Normal	47/81 (58.0%)	37/45 (82.2%)	0.047
WCH/Pre-hypertension	7/81 (8.6%)	2/45 (4.4%)	
Masked hypertension	21/81 (25.9%)	4/45 (8.9%)	
Ambulatory/severe ambulatory hypertension	6/81 (7.4%)	2/45 (4.4%)	
Prevalence of hypertension	27/81 (33.3%)	6/45 (13.3%)	0.019

CSK, congenital solitary kidney; CAKUT, congenital abnormalities of kidney and urinary tract; WCH, white coat hypertension

#### Reduced dipping and abnormal load

No statistically significant differences in the percentage of patients with reduced dipping were observed between Group A and Group B: in both groups, we observed a similar high prevalence of reduced systolic dipping (64% and 58%, respectively, *p* = 0.48), while the prevalence of reduced diastolic dipping was lower (17% vs. 18%, respectively, *p* = 0.94).

In the subgroup of 79 patients for whom load values were available, the prevalence of an abnormal systolic load was 47/61 (44.3%) in Group A and 4/18 (22.2%) in Group B (*p* = 0.092), while for an abnormal diastolic load it was 18/61 (29.5%) in Group A and 3/18 (16.7%) in Group B (*p* = 0.278).

### Risk factors

An analysis of potential risk factors for hypertension was performed in Group A, but no association was found between any of these and the presence of hypertension (Table 5).

**Table 5:** Analysis of risk factors for hypertension (ambulatory/severe ambulatory or masked hypertension) in 81 patients with CSK (Group A)

	Hypertension		*p* value
	Yes	No	
Age, median, years	11.3	12.5	0.822
Male sex, %	81.5	72.2	0.423
Obese, %	7.4	3.7	0.597
Preterm < 37 weeks, %	15.8	17.9	0.859
Gestational age, median, weeks	38	39	0.268
Low birth weight < 2500 g, %	21.1	13.5	0.470
Birth weight, median, grams	3060	3150	0.463
Vesico-ureteric reflux, %	5.9	24.2	0.141
Compensatory renal hypertrophy, %	92.6	87.0	0.710
eGFR, median, ml/min/1.73m^2^	122.1	123.4	0.270
Hyperfiltration %	22.2	33.3	0.302

CSK, congenital solitary kidney; eGFR, estimated glomerular filtration rate

## Discussion

We evaluated ABPM results and compared them with OBP in a cohort of 126 children with a developmental abnormality of the kidney and urinary tract, of whom 81 had a CSK and 45 had two kidneys; both cohorts had normal renal parenchyma at scintigraphy, normal estimated glomerular filtration rate, proteinuria, and concentrating capacity.

At office recordings, median BP SDSs were normal in both groups, without significant differences between them. At ABPM, median SDSs, although normal, were significantly higher in children with a CSK than in children with two kidneys: indeed, in the cohort with CSK the distribution of BP was shifted to higher values (Figs. 1 and 2). Moreover, after ABPM was performed, a high percentage of masked hypertension was detected in children with CSK (25.9%), contributing significantly to the global prevalence of hypertension diagnosed in this group of patients and to the identification of children who might need pharmacologic treatment during further follow-up. On the contrary, the prevalence of masked hypertension in Group B was 8.9%, similar to that reported in the general pediatric population, in which it is around 7% [20, 21]. The identification of masked hypertension with ABPM is considered important, as available data show that patients with masked hypertension have, on average, a similar left ventricular mass index as sustained hypertensives [20].

None of the risk factors analyzed in our children with CSK were associated with hypertension. In particular, the frequency of hyperfiltration, one of the main mechanisms associated with hypertension and damage progression in animal models with CSK, was comparable in normotensive and hypertensive subjects with CSK (33.3% vs. 22.2%, *p* = 0.302) in our cohort. Interestingly, vesico-ureteric reflux was four times more frequent in our cohort in children with CSK showing normal BP than in those with hypertension (24.0% vs. 5.9%), even if the difference was not statistically significant; moreover, although we found no association between the presence of renal hypertrophy and BP, it should be remembered that all kidneys were normal at scintigraphy.

Since CSK originates from an embryological disorder, there may be subtle dysplastic changes, not evident on imaging or by standard laboratory assessment, favoring hypertension. Notwithstanding, in our series comparing two groups of children with a developmental disorder of the kidney and urinary tract, CSK appeared to contribute per se to hypertension. In spite of the high variability of nephron numbers in the population [22], we believe that the reasonable explanation for this effect is that in CSK the number of nephrons is likely to be lower than in children with two kidneys. A reduced number of nephrons has been previously demonstrated as one of the factors contributing to the development of primary hypertension [3]. Therefore, our comparison between children with a CSK and children with other types of CAKUT strengthens the previously recognized notion of an increased risk of hypertension in children with a CSK.

A comparison between our data and previous studies is difficult to make due to the differences in populations, definitions of hypertension, and outcomes. In particular, many studies combined data from children with both congenital and acquired solitary kidneys, comparing them with the normal population. To our knowledge, our study is the first comparing children with a CSK with a population presenting with CAKUT and two kidneys.

In 44 children with CSK, Dursun et al. found mean BP values to be similar to those of normal controls, but the prevalence of hypertension in CSK was higher (23% vs. 5%). They also observed an inverse correlation between kidney length and ABPM MAP and loads [7].

Westland et al. compared results obtained with office BP and with ABPM in 28 children with CSK, and also found systolic, diastolic, and MAP SDSs within normal limits; however, children with CSK had higher mean ABPM SDSs than patients with acquired solitary kidney, except for nocturnal parameters. Whereas OBP identified 7% of hypertensive children, ABPM revealed 24-h hypertension in 25%, therefore unmasking 18% of subjects with masked hypertension [8].

In sheep, a species in which nephrogenesis is complete before birth, as in humans, CSK is associated with a reduced capacity to maintain an adequate sodium excretion. It is theorized that impaired sodium excretion together with glomerular hyperfiltration exposes subjects with CSK to the risk of a progressive increase in arterial pressure and loss of renal function with aging [23]. The dysregulation of BP mainly for systolic values in our cohort should be consistent with the hypothesis of extracellular fluid expansion [23]**.** Therefore, avoiding excessive salt intake, which is a common characteristic of Western diets and is implicated in cardiovascular disease, appears to be indicated from childhood in children with CSK, as also highlighted in two recent reviews [24, 25].

Our study has some limitations. Because of its retrospective nature, there could be some bias in the selection of our patients: firstly, because they were followed in tertiary centers, secondly, because ABPM was not performed systematically in all patients with CAKUT. However, we believe that this potential bias is in some way controlled by the fact that our analysis only included patients with normal renal parenchyma at scintigraphy and normal renal function at standard laboratory assessment. Another limit is that performing a second ABPM in abnormal cases could mitigate the high proportion of masked hypertension, as demonstrated in other follow-up studies [21]. A third limitation is represented by incomplete data on load.

## Conclusions

Data from our study confirm that a CSK can be associated with BP increase from childhood. Therefore, children with CSK, even if morphologically and functionally normal at standard laboratory and imaging assessment, deserve long-term follow-up and periodic BP evaluation, also with ABPM, which has proved to be of value in the screening of hypertension in CSK.

## Electronic supplementary material

ESM 1 (DOCX 16 kb).

## References

[1] Shimamura T, Morrison AB (1975). A progressive glomerulosclerosis occurring in partial five-sixths nephrectomy. Am J Pathol.

[2] Luyckx VA, Brenner BM (2010). The clinical importance of nephron mass. J Am Soc Nephrol.

[3] Keller G, Zimmer G, Mall G, Ritz E, Amann K (2003). Nephron number in patients with primary hypertension. N Engl J Med.

[4] Schreuder MF, Langemeijer ME, Bökenkamp A, Delemarre-Van da Waal HA, Van Vijk JAE (2008). Hypertension and microalbuminuria in children with congenital solitary kidneys. J Paediatr Child Health.

[5] La Scola C, Ammenti A, Puccio G, Lega MV, De Mutiis C, Guiducci C, De Petris L, Perretta R, Venturoli V, Vergine G, Zucchini A, Montini G (2016). Congenital solitary kidney in children: size matters. J Urol.

[6] Mei-Zahav M, Korzets Z, Cohen I, Kessler O, Rathaus V, Wolach B, Pomeranz A (2001). Ambulatory blood pressure monitoring in children with a solitary kidney- a comparison between unilateral renal agenesis and uninephrectomy. Blood Press Monit.

[7] Dursun H, Bayazit AK, Cengiz N, Seydaoglu G, Buyukcelik M, Soran M, Noyan A, Anarat A (2007). Ambulatory blood pressure monitoring and renal functions in children with a solitary kidney. Pediatr Nephrol.

[8] Westland R, Schreuder MF, van der Lof DF, Vermeulen A, Dekker-van der Meer IMJ, Bökenkamp A, van Vijk JAE (2014). Ambulatory blood pressure monitoring is recommended in the clinical management of children with a solitary functioning kidney. Pediatr Nephrol.

[9] Shirzai A, Yildiz N, Biyikli N, Ustunsoy S, Benzer M, Alpay H (2014). Is microalbuminuria a risk factor for hypertension in children with solitary kidney?. Pediatr Nephrol.

[10] Tabel Y, Aksoy Ö, Elmas AT, Çelik SF (2015). Evaluation of hypertension by ambulatory blood pressure monitoring in children with solitary kidney. Blood Press.

[11] Seeman T, Patzer L, John U, Dusek J, Vondràk K, Janda J, Misselwitz J (2006). Blood pressure, renal function, and proteinuria in children with unilateral renal agenesis. Kidney Blood Press Res.

[12] Flynn JT, Kaelber DC, Baker-Smith CM, Blowey D, Carroll AE, Daniels SR, de Ferranti SD, Dionne JM, Falkner B, Flinn SK, Gidding SS, Goodwin C, Leu MG, Powers ME, Rea C, Samuels J, Simasek M, Thaker VV, Urbina EM (2017). Subcommittee on screening and Management of High Blood Pressure in children. Clinical practice guideline for screening and Management of High Blood Pressure in children and adolescents. Pediatrics.

[13] Flynn JT, Daniels SR, Hayman LL, Maahs DM, McCrindle BW, Mitsnefes M, Zachariah JP, Urbina EM (2014). Update: ambulatory blood pressure monitoring in children and adolescents: a scientific statement from the American Heart Association. Hypertension.

[14] Baumgart P, Kamp J (1998). Accuracy of the Spacelabs medical 90217 ambulatory blood pressure monitor. Blood Press Monit.

[15] Wühl E, Witte K, Soergel M, Mehls O, Schaefer F, for the German Working Group on Pediatric Hypertension (2002). Distribution of 24-h ambulatory blood pressure in children: normalized reference values and role of body dimensions. J Hypertens.

[16] Cole TJ, Green PJ (1992). Smoothing reference centile curves: the LMS method and penalized likelihood. Stat Med.

[17] Dinkel E, Ertel M, Dittrich M, Peters H, Berres M, Schulte-Wissermann H (1985). Kidney size in childhood. Sonographical growth charts for kidney length and volume. Pediatr Radiol.

[18] Hellerstein S, Chambers L (2008). Solitary kidney. Clin Pediatr.

[19] R Core Team. R (2016). A language and environment for statistical computing.

[20] Verberk WJ, Kessels AG, de Leeuw PW (2008). Prevalence, causes, and consequences of masked hypertension: a meta-analysis. Am J Hypertens.

[21] Lurbe E, Torro I, Alvarez V, Nawrot T, Paya R, Redon J, Staessen JA (2005). Prevalence, persistence, and clinical significance of masked hypertension in youth. Hypertension.

[22] Bertram JF, Douglas-Denton RN, Diouf B, Hugson MD, Hoy WE (2011). Human nephron number: implications for health and disease. Pediatr Nephrol.

[23] Lankadeva YR, Singh RR, Tare M, Moritz KM, Denton KM (2014). Loss of a kidney during fetal life: long term consequences and lessons learnt. Am J Physiol Renal Physiol.

[24] Schreuder MF (2018). Life with one kidney. Pediatr Nephrol.

[25] Cochat P, Febvey O, Bacchetta J, Bérard E, Cabrera N, Dubourg L (2019). Towards adulthood with a solitary kidney. Pediatr Nephrol.

