# Supplementary material for: Born with a solitary kidney: at risk of hypertension
Source: Pediatr Nephrol. 2020 Mar 24;35(8):1483–90. doi: 10.1007/s00467-020-04535-1 (PMC7316689; doi:10.1007/s00467-020-04535-1)
Supplement: Supplementary file 1 — (DOCX 16 kb). [file 467_2020_4535_MOESM1_ESM.docx]

**Online Resource 1: Distribution of blood pressure classes according to American Heart Association staging in the subset of 79 patients for whom Load values were available. Unclassified patients are shown as a separate category**

|  | **Group A** | **Group B** | ***p* value** |
| --- | --- | --- | --- |
| Severe hypertension | 4/61 (6.6%) | 1/18 (5.6%) | **0.541** |
| Hypertension | 1/61 (1.6%) | 0/18 (0) |  |
| Masked hypertension | 13/61 (21.3%) | 2/18 (11.1%) |  |
| White coat hypertension | 4/61 (6.6%) | 0/18 (0) |  |
| Pre-hypertension | 1/61 (1.6%) | 0/18 (0) |  |
| Normal | 25/61 (41.0%) | 13/18 (72.2%) |  |
| Unclassified | 13/61 (21.3%) | 2/18 (11.1%) |  |
|  |  |  |  |
| Prevalence of hypertension | 18/48 (37.5%) | 3/16 (18.7%) | **0.225** |

**Comment:** it should be observed that the classification of the American Heart Association

leaves some cases unclassified: in our sample 15 cases (19%). So, unclassified patients seem to be a rather common event with the classification that includes load values.
